# Nuclear localized Influenza nucleoprotein N-terminal deletion mutant is deficient in functional vRNP formation

**DOI:** 10.1186/1743-422X-11-155

**Published:** 2014-08-31

**Authors:** Abel Sanchez, Christian F Guerrero-Juarez, Jose Ramirez, Laura L Newcomb

**Affiliations:** Department of Biology, California State University San Bernardino, 5500 University Parkway, San Bernardino, CA 92407 USA

**Keywords:** Influenza, Virus, RNA, vRNP, Nucleoprotein

## Abstract

**Background:**

The influenza RNA dependent RNA polymerase synthesizes viral RNA in the nucleus as functional viral ribonucleoprotein (vRNP) complexes with RNA and nucleoprotein (NP). The N-terminus of NP contains an unconventional nuclear localization signal (NLS) important for initial vRNP nuclear localization but which also interacts with various host factors.

**Methods:**

To study the role of the N-terminus of NP aside from NLS function, we generated an N-terminal NP deletion mutant, del20NLS-NP, encoding the conventional SV40 T-antigen NLS in place of the first 20 amino acids of NP. We characterized expression, location, and activity of del20NLS-NP compared to wild type NP using reconstituted vRNP assays, cellular fractionation, Western blotting, and reverse transcription-PCR. We assessed NP nucleotide binding with gel-shift assays and analyzed NP complexes using 1D blue native gel electrophoresis.

**Results:**

del20NLS-NP is expressed, localized in the nucleus and cytoplasm, and maintains ability to bind nucleic acids. Despite this, del20NLS-NP exhibits a defect in viral RNA expression exacerbated by increasing vRNA template length. We find diminished del20NLS-NP high molecular weight complexes in protein extracts; evidence the defect is with functional vRNP formation. Interestingly, the shortest template, NS vRNA, exhibits a limited defect. However, this is not due to short template size, but rather activity of the NS protein(s). Expression of NS1 rescues the gene expression defect primarily at the protein level, a finding consistent with the known role of NS1 as a viral mRNA translational enhancer. NS1 mutant analysis confirms NS1-RNA binding is not required for the translational enhancement and reveals the NS1-CPSF30 interaction surface is essential.

**Conclusions:**

del20NLS-NP is a nuclear localized NP mutant able to bind nucleic acids but inefficient for assembly of functional vRNPs inside the host cell. Our results add to growing evidence the N-terminus of NP plays important roles aside from vRNP nuclear localization. We demonstrate the utility of this partially functional NP mutant to characterize the influence of additional proteins on viral gene expression. Our studies reveal the NS1-CPSF30 interaction surface is required for the ability of NS1 to enhance viral protein translation, supporting a function for this NS1 domain in the cytoplasm.

## Introduction

Influenza A viruses cause a highly contagious respiratory infection, and both seasonal and pandemic strains remain a persistent health issue for humans. Influenza pandemics occurred three times in the 20th century (1918, 1957 and 1968) and in 2009 the novel H1N1 influenza (swine flu) became the first influenza pandemic of the 21st century. Equally troubling are the continued human infections with the highly pathogenic H5N1 avian influenza (bird flu) in Asia, Europe, and Africa. This subtype has not yet gained the ability to readily transmit from human to human, but research has demonstrated this virus is able to evolve increased transmissibility in the ferret model [1, 2]. The lack of preparation for the rapidly spreading novel H1N1 and continued threats of a possible avian influenza outbreak make it painfully evident that more knowledge of the basic molecular activities required for influenza replication is needed so that new antiviral targets can be identified and therapies developed.

Influenza is a negative strand RNA virus with eight vRNA segments. The influenza RNA dependent RNA polymerase (RdRP) is a three-subunit complex comprised of PA, PB1, and PB2, which transcribes and replicates viral RNA in the nucleus. The viral RdRP binds an RNA panhandle formed by complimentary interaction between the 5’ and 3’ ends of the viral genome [3, 4]. NP encapsidates each vRNA template and also makes direct protein contacts with the viral RdRP [5]. Together, the viral RdRP, NP and each vRNA segment form functional viral ribonucleoprotein complexes (vRNPs) responsible for transcription and replication of the viral genome in the nucleus of the host cell.

The mechanisms of transcription by the viral RdRP are well characterized. Influenza viral mRNAs are similar to host messages as they contain both a 5’ cap and 3’ polyA tail, but these modifications are not acquired through host cellular processing. The 5’ cap is stolen from nascent cellular mRNAs via a ‘cap-snatching’ mechanism [6]. The influenza RdRP interacts with host RNA polymerase II C-terminal domain [7], placing the viral RdRP within the environment of nascent cellular mRNAs, which acquire the 5’ cap modification early during transcription. The PB2 subunit of the influenza RdRP recognizes and binds cellular capped mRNAs [8] while the PA subunit uses its intrinsic endonuclease activity to cleave the 5’ capped RNA [9, 10]. The resulting cellular capped RNA fragment is used by the PB1 subunit of the influenza RdRP as primer for polymerization and viral mRNA synthesis. Polyadenylation of the 3’ end of the viral mRNA occurs by a ‘stuttering’ mechanism, wherein the viral RdRP slips along five to seven uracil residues approximately 17 to 22 nucleotides from the 5' end of each vRNA template, resulting in the polymerization of a poly A tail [11, 12]. Influenza gene expression further requires the splicing of two viral mRNAs and the nuclear export of spliced mRNAs (NS2 and M2), intron containing mRNAs (NS1 and M1), and intronless mRNAs (NP,PA, PB1, PB2, HA, and NA). Evidence suggests a model wherein the resident RdRP of the vRNP is responsible for transcription, while a soluble RdRP is responsible for replication from the vRNA template [13].

NP does not directly participate in the transcriptional activities described above, but NP is essential for efficient transcription in the host cell as part of the functional vRNP. Replication actively requires NP as NP encapsidates both the vRNA and cRNA replication products, and NP is required for synthesis of template sized RNA *in vitro* [14]. However, NP is more than a structural RNA binding protein. NP is intricately involved in promoting viral RNA replication: NP is required for anti-termination at the polyA addition site during vRNA to cRNA replication [15], NP and the RdRP stabilize nascent cRNA and vRNA replication products [16], and although NP is not essential for RNA replication on short templates [17], NP protein interaction with the viral RdRP enhances unprimed RNA initiation *in vitro* [18]. The cRNA is then used as template to produce more vRNA. Viral RNA replication leads to increased vRNA templates available fo transcription, amplifying production of viral mRNAs. Thus, in addition to coating the RNA template, NP plays an integral role in viral RNA replication and proper viral gene expression.

NP is a multi-functional protein that interacts with a number of viral and host proteins at various times during infection (for review see [19]). The N-terminus of NP contains an unconventional nuclear localization signal at amino acids 3-13 (nNLS- SxGTKRSYxxM) critical for both NP and vRNP nuclear localization [20, 21]. Recombinant virus encoding mutations within the unconventional NLS were attenuated with NP localized primarily to the cytoplasm, resulting in decreased viral gene expression [22]. The N-terminus also interacts with host RNA processing factor UAP56, as NP amino acids 1-20 were sufficient to bind UAP56 [23]. UAP56 is a member of the DEAD-box family of RNA dependent ATPases and RNA helicases [24], and is involved in cellular mRNA remodeling during mRNA processing and nuclear export [25]. Experiments with purified NP and UAP56 proteins *in vitro* indicate that UAP56 functions as a chaperone to promote free NP binding to nascent viral RNA replication products resulting in enhanced viral RNA synthesis [23, 26]. The goal of this study was to address the role of N-terminal NP interactions in the context of the host cell. To accomplish this, we designed a plasmid encoding the conventional NLS from SV40 T-antigen (PKKKRKV) in place of the N-terminal 20 amino acids of NP (del20NLS-NP).

We report here the characterization of del20NLS-NP using transfection to express reconstituted vRNPs in human embryonic kidney cell line (293 T). We find that del20NLS-NP is expressed, localized, and binds nucleotides as wild type NP (WT-NP). However, RNA expression from influenza vRNA templates in the presence of del20NLS-NP is significantly decreased compared to WT-NP. Increasing vRNA template length exacerbates the RNA expression defect. To assess vRNP formation we analyzed cell protein extracts for NP containing high molecular weight complexes using 1D blue native gel electrophoresis and found substantial decrease in high molecular weight complexes containing del20NLS-NP compared to WT-NP. These results contribute evidence that aside from the importance for nuclear localization, the N-terminus of NP is required for efficient formation of vRNPs.

## Results and discussion

### del20NLS-NP is expressed, localized, and binds nucleic acids as WT-NP

**Figure 1:**
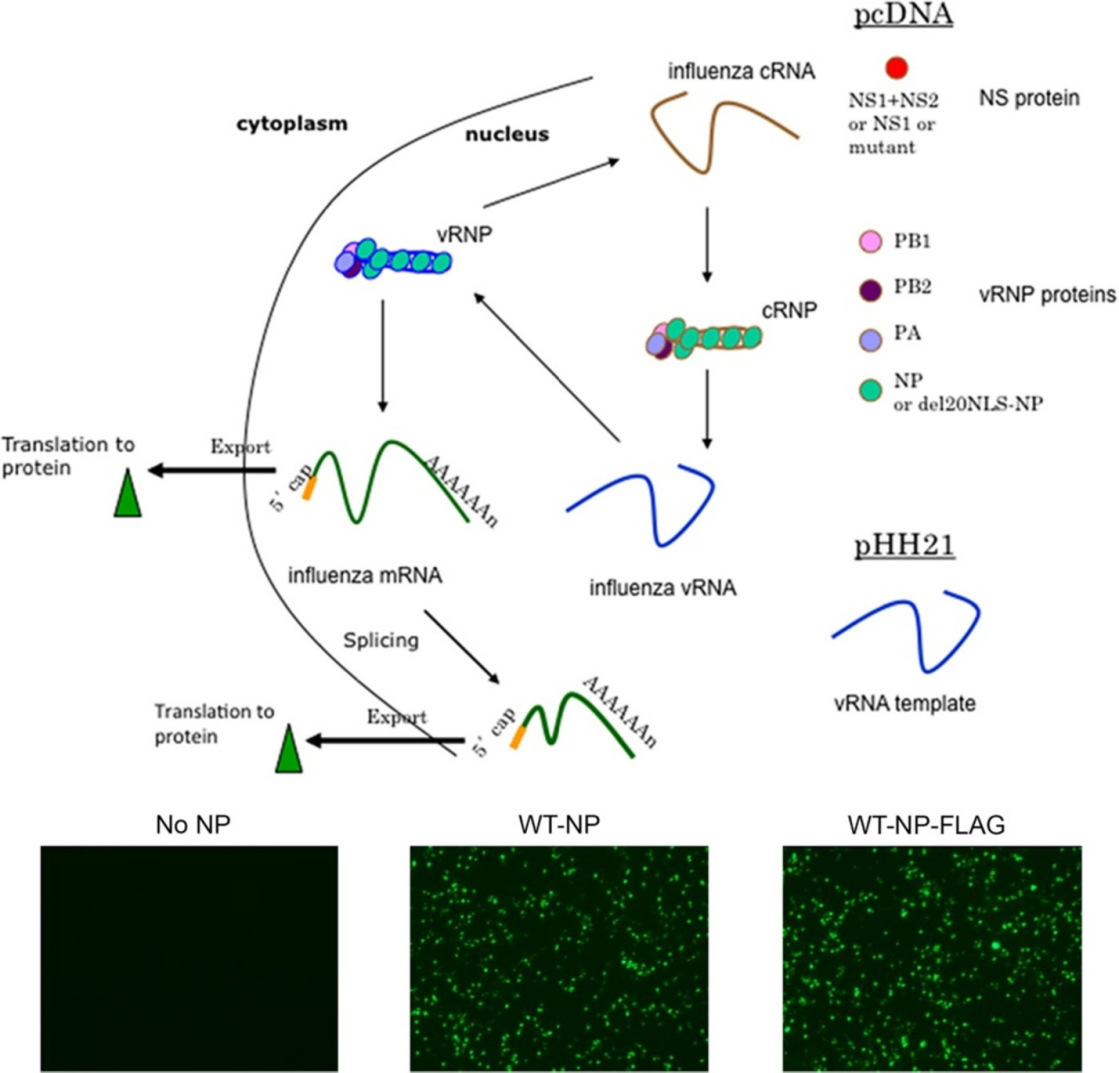
**Reconstituted vRNPs.** Cellular RNA polymerase II drives expression from pcDNA plasmids to produce 5’ capped and 3’ polyadenylated mRNAs that are translated to express the proteins components of the influenza vRNP and NS proteins as described for each experiment. Cellular RNA polymerase I drives expression from pHH21 plasmids to produce unmodified full-length influenza vRNAs. Although influenza contains 8 different vRNA segments to form a complete virus, here one vRNA segment is expressed so that virus is not generated. Bottom panels illustrates WT-NP-FLAG is as functional as WT-NP in reconstituted vRNP assays using GFP-M vRNA template.

To address the role of N-terminal NP interactions inside the host cell, we set out to construct a plasmid encoding an N-terminal NP deletion mutant able to localize to the nucleus, where viral RNA synthesis and processing occur. The N-terminal 20 amino acids of NP are sufficient for interaction with host RNA processing factor UAP56 [23], but these first 20 amino acids of NP also include an important, unconventional nuclear localization signal (NLS) [20–22]. To ensure sufficient nuclear localization of mutant NP proteins, we generated a plasmid to encode the conventional NLS from SV40 T-antigen (PKKKRKV) at the N-terminus of NP in place of the N-terminal 20 amino acids (del20NLS-NP). We also engineered a FLAG epitope tag at the C-terminus of both WT-NP and del20NLS-NP for ease of detection and immuno-purification. To ensure the epitope FLAG tag did not alter NP function in the context of reconstituted vRNPs, we confirmed WT-NP-FLAG was as functional as untagged WT-NP for viral gene expression (GFP-M) in our assay (Figure 1).

**Figure 2:**
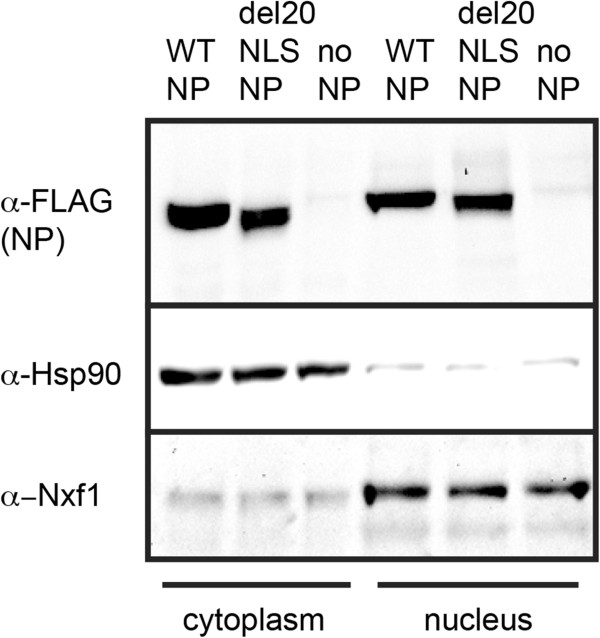
**Mutant del20NLS-NP is expressed and localized in the nucleus and cytoplasm as WT-NP.** 293 T cells were transfected with plasmid mixes to express reconstituted vRNPs containing influenza RdRP with vRNA templates and either WT-NP, del20NLS-NP, or vector control as indicated. Cells were fractionated and protein extracts from both the cytoplasm and nucleus were analyzed by western blot. Anti-FLAG antibody detects the C-terminal epitope tag of both WT-NP and del20NLS-NP. Hsp90 is a protein localized to the cytoplasm while Nxf1 is localized to the nucleus, demonstrating cellular fractionation with only slight cross contamination of cellular fractions. Shown is a representative experiment, repeated more than three times.

To assess expression, localization, and activity of del20NLS-NP, we transfected human embryonic kidney cell line (293 T) with plasmids to express reconstituted vRNPs comprised of the viral vRNA template, viral RdRP, and either WT-NP, del20NLS-NP, or no NP (vector) negative control. Expression of the vRNA template was driven by host RNA polymerase I (pHH21 DNA plasmids), while expression of mRNAs encoding the viral RdRP subunits and NP were driven by host RNA polymerase II (pcDNA DNA plasmids), similar to the system used for expressing the eight vRNPs required to generate recombinant virus [27], except only one vRNA segment is expressed at a time in the host cell so no virus is generated (Figure 1). Nuclear and cytoplasmic proteins were isolated 48 hours post transfection. Western blotting with anti-FLAG antibody confirmed del20NLS-NP was expressed and present in both the nucleus and cytoplasm, similar to WT-NP (Figure 2). Hsp90 and Nxf1 served as cytoplasmic and nuclear localized and loading controls, respectively. Together, the control proteins demonstrated near purity of the cellular fractionation and allowed confirmation that del20NLS- NP was expressed and present in the nucleus as WT-NP (Figure 2). This confirms sufficient del20NLS-NP is present in the nucleus where viral RNA synthesis and processing occur to allow analysis of N-terminal NP nuclear functions aside from nuclear import.

**Figure 3:**
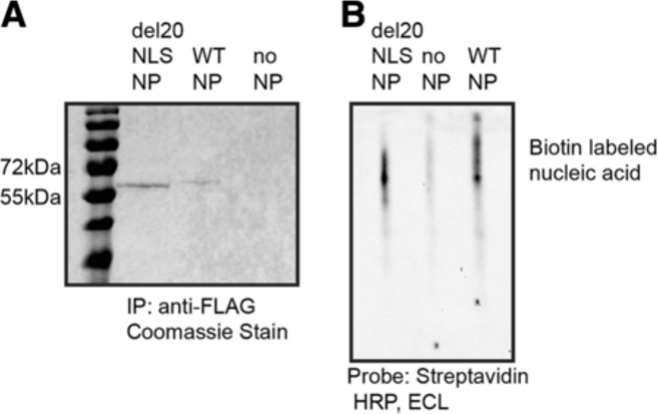
**Mutant del20NLS-NP maintains nucleic acid binding as WT-NP. A**. 293 T cells were transfected with plasmids to express WT-NP, del20NLS-NP, or vector control as indicated. Total protein extracts were isolated and subject to immuno purification with anti-FLAG antibody and elution with FLAG peptide. Elutant was separated via SDS-PAGE gel and stained with Coomassie blue. **B**. Purified NP proteins incubated with biotin labeled single stranded DNA were resolved via native PAGE gel, transferred to nitrocellulose membrane, and probed with Streptavidin-HRP. Purified del20NLS-NP and WT-NP shift the biotin labeled ssDNA as Streptavidin-HRP is detected with ECL reagents. Unbound biotin labeled ssDNA was not detected. Shown is a representative experiment, repeated in triplicate.

To ensure that del20NLS-NP maintains RNA binding activity, we carried out gel shift analysis with immuno purified NP proteins and biotin labeled single stranded DNA (ssDNA). The crystal structure of NP reveals a highly positively charged nucleic acid binding groove for interaction with viral RNA, which should not be disturbed by deletion of the N-terminal 20 amino acids [28]. NP RNA binding is blocked by saturation with single stranded DNA [18]; therefore we incubated the purified NP proteins with biotin labeled ssDNA corresponding to the 5’ and 3’ ends of the viral RNA. Coomassie blue stain of the immuno purified elutant separated by SDS-PAGE showed no detectable contaminating proteins (Figure 3). Both purified WT-NP and del20NLS-NP, but not the no NP control, shift the biotin labeled ssDNA, confirming that del20NLS-NP maintains nucleic acid binding activity as expected (Figure 3). Unbound ssDNA was consistently not detected in our no NP negative control; it is probable unbound ssDNA does not transfer and stay bound to nitrocellulose membranes as well as that associated with protein under our assay and detection conditions.

### del20NLS-NP is deficient in RNA expression from vRNA templates

**Figure 4:**
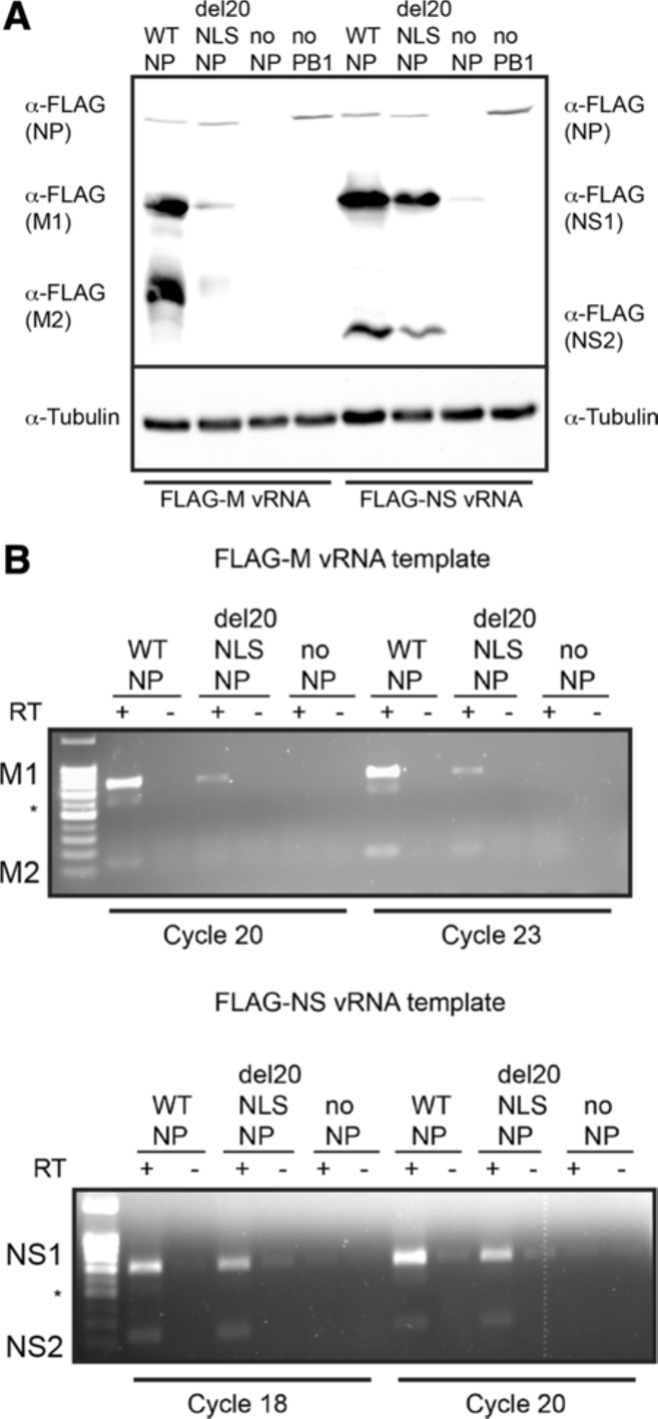
**Mutant del20NLS-NP is severely defective for gene expression from M vRNA but less so from NS vRNA.** 293 T cells were transfected with plasmid mixes to express reconstituted vRNPs containing influenza RdRP with either WT-NP, del20NLS-NP, or no NP, and either FLAG-NS vRNA or FLAG-M vRNA template, as indicated. **A**. Total protein was separated by SDS-PAGE and analyzed by Western blot. Reconstituted vRNP with WT-NP but no PB1 protein serves as an additional negative control. Anti-FLAG antibody detects NP-FLAG and FLAG-NS1, FLAG- NS2, FLAG-M1, and FLAG-M2 proteins. Tubulin serves as loading control. Shown is a representative experiment, repeated more than three times. **B**. Total RNA was isolated, DNase treated, and 1 μg subject to reverse transcription with oligo dT and PCR with gene specific primers for M and NS as indicated. Asterisks represents aberrant product observed with FLAG sequence containing templates. Shown is a representative experiment, repeated in triplicate.

To investigate the function of del20NLS-NP, we first examined gene expression from reconstituted vRNPs containing either NS vRNA (853 nucleotides) or M vRNA (1005 nucleotides) templates. The viral NS and M segments express both intron containing mRNAs, encoding NS1 and M1 proteins, and spliced mRNA counterparts, encoding NS2 (NEP) and M2 proteins, allowing us to examine gene expression and mRNA processing by splicing in the presence of del20NLS-NP. FLAG N-terminal tags were fused to the NS and M genes to readily observe gene expression from the vRNA templates. Our results revealed that del20NLS-NP vRNPs result in a significant decrease in FLAG-M1 and FLAG-M2 protein expression, while FLAG-NS1 and FLAG-NS2 protein expression was only slightly decreased compared to the expression levels with WT-NP vRNPs (Figure 4A). Identical results were observed with the untagged M and NS vRNA templates (data not shown). Reverse transcription with oligo dT and semi-quantitative PCR with gene specific primers demonstrated the gene expression defect resides at the level of viral mRNA expression. As with protein expression, FLAG-M mRNA products were severely decreased in the presence of del20NLS-NP while FLAG-NS mRNA products were decreased, but to a lesser extent (Figure 4B). Our results show no defect in splicing in the presence of del20NLS-NP as both FLAG-NS2 and FLAG-M2 protein and RNA products were detected (Figure 4).

### Gene expression defect is not restricted to M vRNA templates

**Figure 5:**
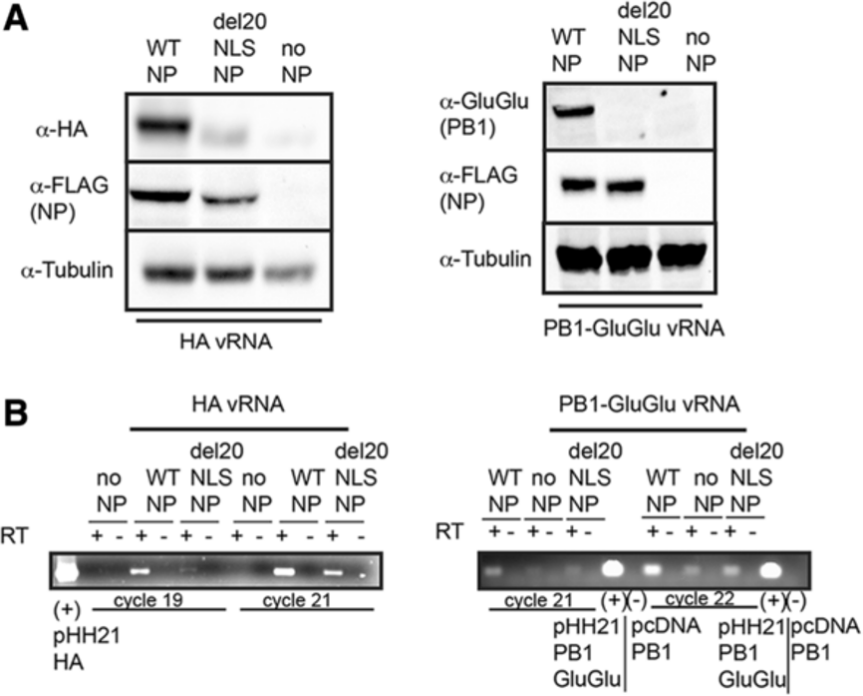
**Mutant del20NLS-NP defect is not specific to the M segment.** 293 T cells were transfected with plasmid mixes to express recombinant vRNPs containing influenza RdRP with either WT-NP, del20NLS-NP, or no NP, and either HA vRNA or PB1-GluGlu vRNA template, as indicated. **A**. Total protein was separated by SDS-PAGE and analyzed by Western blot. Anti-FLAG antibody detects NP FLAG, anti-HA detects HA, anti-GluGlu detects PB1-GluGlu, and anti-tubulin detects tubulin and serves as loading control. **B**. Total RNA was isolated, DNase treated, and 1 μg subject to reverse transcription with oligo dT and PCR with gene specific primers for HA and PB1-GluGlu; pHH21-HA and pHH21 PB1-GluGlu DNA plasmids serve as positive controls while pcDNA PB1 untagged DNA plasmid serves as negative control, in addition to no RT. Shown is a representative experiment, repeated in triplicate.

We first addressed whether or not the severe defect was restricted to M expression. We found that both HA vRNA (1778 nucleotides), a late expressed gene encoding an external protein, and PB1-Glu-Glu vRNA (2333 nucleotides), an early expressed gene encoding an internal protein, were also severely defective for protein and RNA expression in the presence of del20NLS-NP (Figure 5). The PB1 Glu-Glu is not functional in reconstituted vRNP assays (unpublished observation, LLN) and thus cannot contribute to vRNP activity. Although our detection methods for each gene utilized different antibodies and PCR primers, the severity of the defect appeared variable; no PB1 protein or RNA was detected with the PB1-Glu-Glu vRNA template in the presence of del20NLS-NP above no NP negative control, while HA protein and RNA were detected with the HA vRNA template, albeit still at much reduced expression compared to WT-NP.

### Increasing vRNA template length exacerbates the defect in viral RNA expression observed in the presence of del20NLS-NP

**Figure 6:**
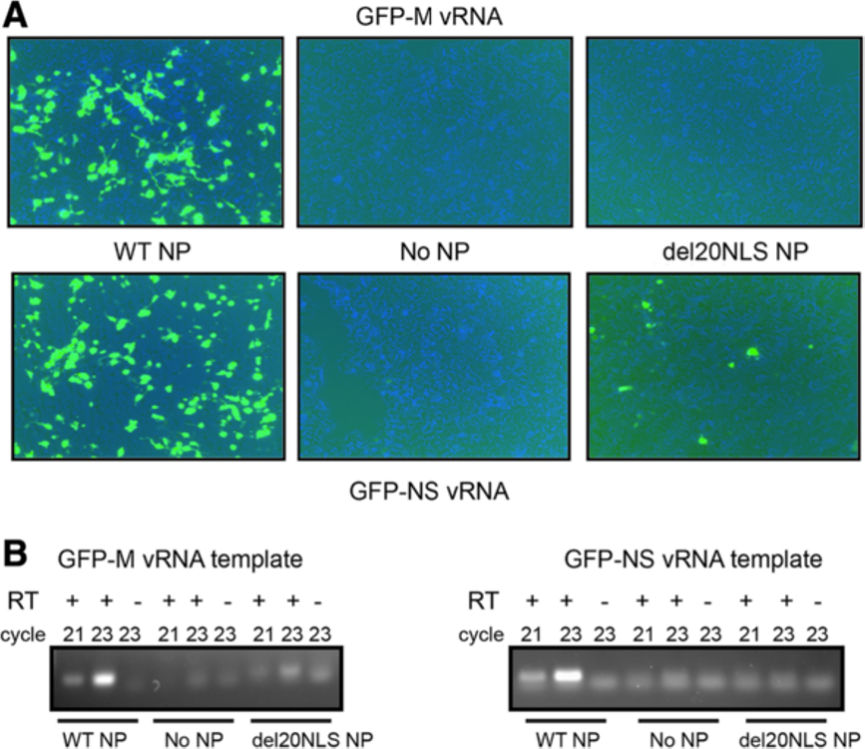
**Mutant del20NLS-NP defect is exacerbated as vRNA template length is increased.** 293 T cells were transfected with plasmid mixes to express recombinant vRNPs containing influenza RdRP with either WT-NP, del20NLS-NP, or no NP, and either GFP-M vRNA (top panels) or GFP-NS vRNA (bottom panels) template, as indicated. **A**. GFP protein was visualized with a Nikon Eclipse TS100 (Nikon Intensilight C-HGFI for fluorescence). **B**. Total RNA was isolated, DNase treated, and 1 μg subjected to reverse transcription with oligo dT and PCR with gene specific primers for M and NS. Shown is a representative experiment, repeated in triplicate.

Since the severity of the influenza RNA expression defect in the presence of del20NLS-NP appeared variable with different sized templates, we decided to directly address the role of template length. To do this, we increased the vRNA template lengths of M and NS by the addition of GFP. Unlike the FLAG tagged genes, both GFP-M and GFP-NS protein and mRNA expression were severely reduced in the presence of del20NLS-NP (Figure 6), supporting the conclusion the defect is exacerbated as vRNA template length is increased. GFP-M proteins were not detected while GFP-NS proteins were observed in select cells (Figure 6A). It is unclear why select cells show robust GFP-NS expression in the presence of del20NLS-NP while the majority of cells are consistent with the del20NLS-NP defect and express no GFP-NS. We speculate varied expression of a factor in these particular cells lessen the del20NLS-NP defect and allow for functional vRNPs to form. Our results show most cells expressing components for reconstituted vRNPs comprised of del20NLS-NP and GFP-NS vRNA do not express GFP-NS, consistent with a worsening of gene expression with lengthened template in the presence of del20NLS-NP.

However HA vRNA (1778 nucleotides) and GFP-M vRNA (1744 nucleotides) are similar in size yet exhibit different degrees of inhibition of gene expression in the presence of del20NLS-NP; while no GFP-M is detected, some HA is detected (compare Figure 6 with Figure 5). This varied degree of gene expression revealed in the presence of del20NLS-NP are consistent with findings that vRNA segments compete with each other for expression, with relative expression greatly influenced by segment length, but also attributed to specific coding regions and UTR sequences [29]. We conclude that del20NLS-NP results in less viral RNA expression and that while lengthening the vRNA template exacerbates the defect observed, other template characteristics, such as RNA secondary structure, play a role in relative viral gene expression.

### Little high molecular weight complexes are formed with del20NLS-NP

**Figure 7:**
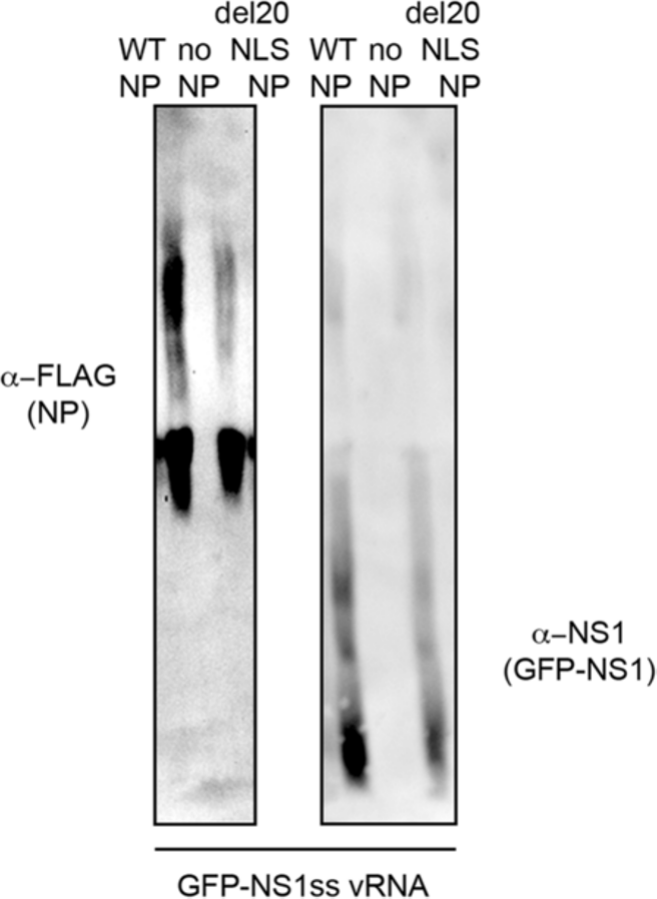
**del20NLS-NP does not form high molecular weight complexes.** 293 T cells were transfected with plasmid mixes to express recombinant vRNPs containing influenza RdRP with either WT-NP, del20NLS-NP, or no NP, and GFP-NS1ss vRNA templates. Total protein extracts were separated on TBE (native) 6% PAGE facilitated by coomassie blue. Proteins were transferred to nitrocellulose and probed with anti-FLAG antibody to detect NP proteins and anti- NS1 antibody to detect NS1 proteins.

To determine if vRNP complexes were formed in the presence of del20NLS-NP, we separated total protein extracts expressing reconstituted vRNPs with GFP-NS1ss (splice site mutation) vRNA templates using 6% 1D Blue Native PAGE to separate large protein complexes [30]. After separation, proteins were transferred to nitrocellulose and the blot probed with anti-FLAG to detect WT-NP and del20NLS-NP. We found a decrease in the amount of NP containing complexes at higher molecular weight with del20NLS-NP compared to WT-NP (Figure 7). To confirm these higher molecular weight complexes seen in the presence of WT-NP were not attributable to NS1 interaction with NP and vRNPs [31–33], we stripped the blot and further probed with anti-NS1. GFP-NS1 is expressed with WT-NP containing vRNPs but less so with del20NLS-NP, as expected, as only some cells are capable of GFP-NS expression in the presence of del20NLS-NP (Figure 6). However, GFP-NS1 is primarily present in lower molecular weight complexes and cannot account for the higher molecular weight complexes containing WT-NP (Figure 7). These results conclude that del20NLS-NP is defective in formation of higher molecular weight complexes seen with WT-NP in cell extracts. These results are consistent with del20NLS-NP deficiency in vRNP formation and conclude a role for the N-terminus of NP independent of the important function for vRNP nuclear localization [21].

### Shortening template length does not alleviate gene expression defect observed in the presence of del20NLS-NP

**Figure 8:**
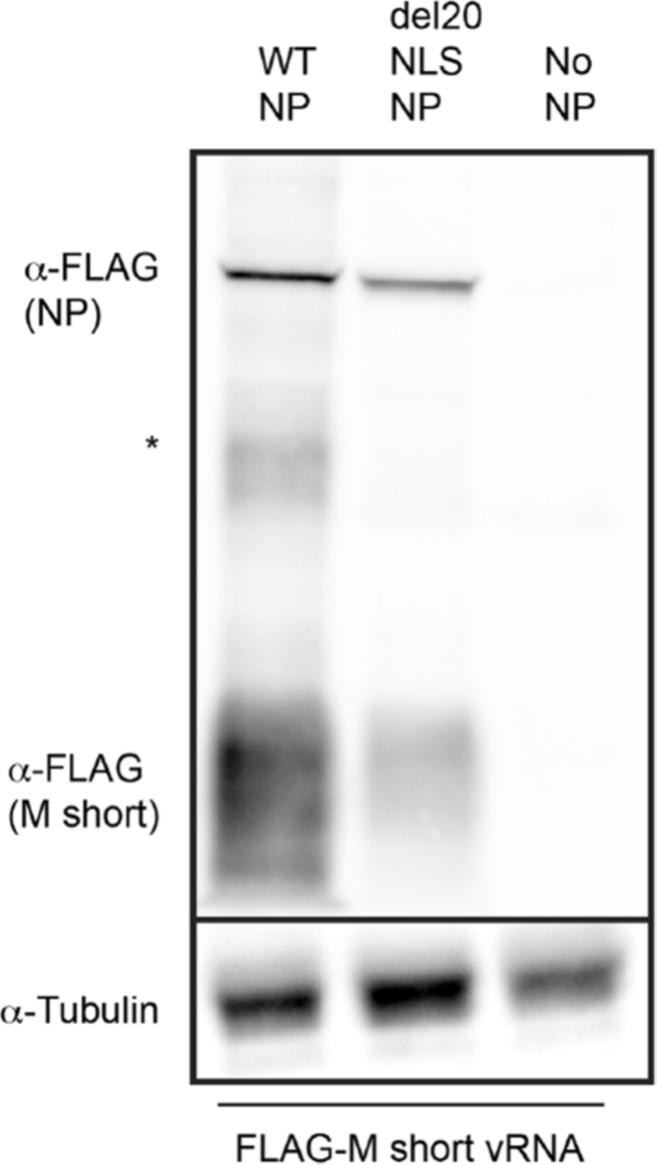
**Gene expression from shortened M vRNA template remains defective in the presence of del20NLS-NP.** 293 T cells were transfected with plasmid mixes to express recombinant vRNPs containing influenza RdRP with either WT-NP, del20NLS-NP, or no NP, and FLAG-M short vRNA template. Total protein was separated by SDS-PAGE and analyzed by Western blot. Anti-FLAG antibody detects NP FLAG and FLAG- M products. Asterisk represents FLAG detected product of unconfirmed identity. Anti-tubulin detects tubulin and serves as a loading control. Shown is a representative experiment, repeated in triplicate.

To determine if template length alone could explain why FLAG-NS was onlyslightly defective for protein expression, and less defective for RNA expression in the presence of del20NLS-NP, we created a shortened FLAG-M template, resulting in a template shorter than NS vRNA. This shortened FLAG-M vRNA template led to robust expression of FLAG-M proteins in the presence of WT-NP but not in the presence of del20NLS-NP (Figure 8). This result rules out the possibility that short template length alone explains why NS protein expression is not inhibited to the same extent as M in the presence of del20NLS-NP.

### Expression of NS1 enhances viral gene expression at the protein level

Our results with the shortened M vRNA template led us to hypothesize that a product(s) of the NS segment compensated for the del20NLS-NP defect. We reasoned that FLAG-NS, with only a small 7 amino acid epitope tag fused to the N-terminus of NS1 and NS2, encoded functional FLAG-NS1 and FLAG-NS2 proteins, while GFP-NS encoded NS proteins that were not functional due to the 233 amino acid GFP sequence fused to the N-terminus of NS1 and NS2 (NEP). We hypothesized that minimal initial expression of FLAG-NS proteins provide activities that result in near normal levels of FLAG-NS protein and increased NS RNA in the presence of del20NLS-NP (Figure 4).

**Figure 9:**
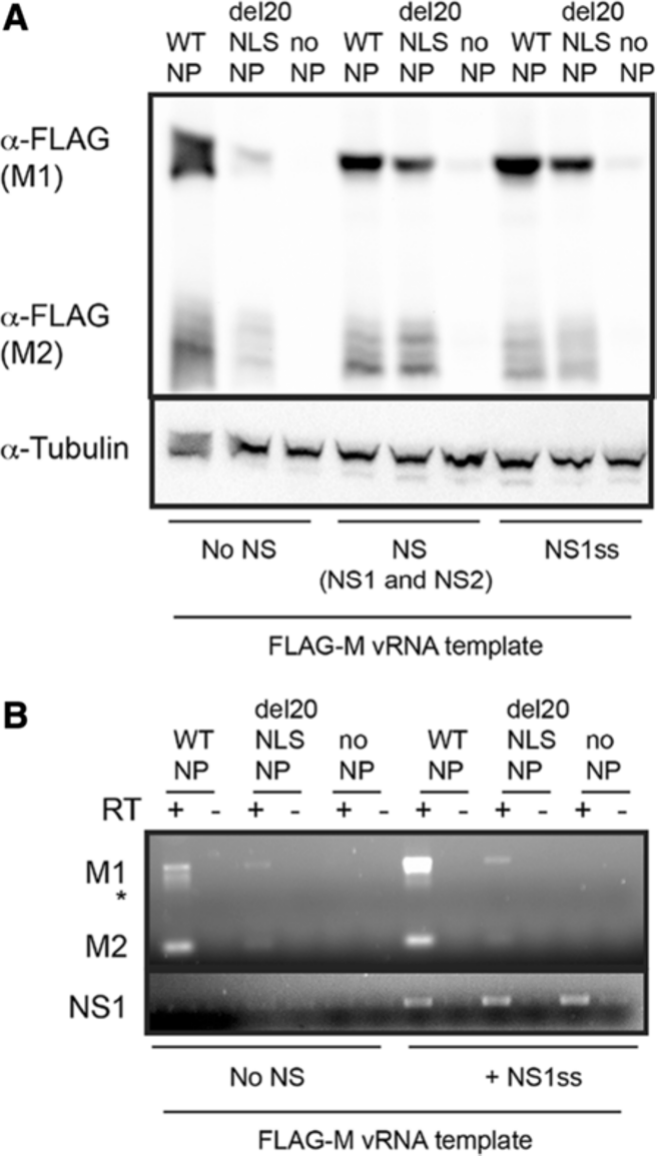
**Expression of NS1 protein rescues gene expression defect observed with mutant del20NLS-NP at protein level.** 293 T cells were transfected with plasmid mixes to express recombinant vRNPs containing influenza RdRP with either WT-NP, del20NLS-NP, or no NP, and FLAG-M vRNA as template in the presence or absence of NS1 and NS2 or NS1 only as indicated. **A**. Total protein was separated by SDS-PAGE and analyzed by Western blotting with anti-FLAG antibody to visualize FLAG-M products. Anti-tubulin detects tubulin and serves as a loading control. **B**. Total RNA was isolated, DNase treated, and 1 μg subject to reverse transcription with oligo dT and PCR for 23 cycles with either M or NS gene specific primers. Asterisk represents aberrant product observed with FLAG-M vRNA templates. Shown are representative experiments, repeated in triplicate.

To test this hypothesis, we performed reconstituted vRNP assays in which the NS1 and NS2 (NEP) proteins were expressed in addition to the components of the vRNP by transfection of pcDNA-NS plasmids to express either NS mRNA which encodes both NS1 and NS2 (NEP) proteins, or NS1ss mRNA encoding a splice site mutation resulting in only NS1 proteins produced (Figure 1). We compared activity of reconstituted vRNPs with FLAG-M vRNA templates in the presence or absence of the NS proteins. As hypothesized, addition of NS1 resulted in increased FLAG-M1 and FLAG-M2 protein expression in the presence of del20NLS-NP (Figure 9A). Consistent with the reported role of NS1 as a viral mRNA translational enhancer [34, 35], NS1 protein improved M expression primarily at the protein level in the presence of del20NLS-NP. RT-PCR revealed FLAG-M mRNA products remain severely reduced in del20NLS-NP samples compared to WT-NP even in the presence of NS1 (Figure 9B).

Interestingly we observed a greater increase in FLAG-M mRNA products in samples expressing NS1 compared to samples without NS1 in the presence of WT-NP but not del20NLS-NP (Figure 9B). NS1 interacts with WT-NP and vRNPs [31–33] and NS1 mutants have altered temporal regulation of influenza RNA expression [36, 37], and therefore it is not surprising that expression of NS1 results in enhanced viral RNA expression. However, our result demonstrates an influence of NS1 in the presence of WT-NP only, suggesting the NS1-NP interaction [33] requires the N-terminus of NP to effect RNA expression. However, because there was no significant increase in FLAG-M mRNA with NS1 in the presence of del20NLS-NP, we attributed the increase in FLAG-M protein expression to the ability of NS1 to enhance viral mRNA translation [34, 35].

**Figure 10:**
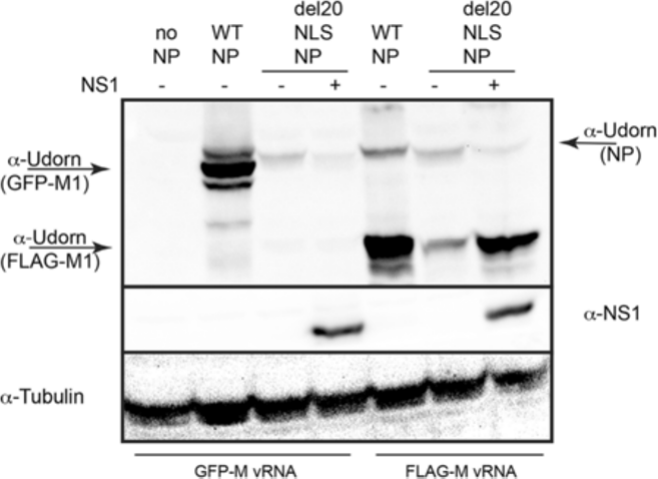
**GFP-M protein expression is not recovered by presence of NS1.** 293 T cells were transfected with plasmid mixes to express recombinant vRNPs containing influenza RdRP with either WT-NP, del20NLS-NP, or no NP, and either GFP-M vRNA or FLAG-M vRNA in the presence or absence of NS1 as indicated. Total protein was separated by SDS-PAGE and analyzed by Western blotting. Anti-Udorn antibody detects NP and M1 products. Anti-NS1 detects NS1. Anti-tubulin detects tubulin and serves as a loading control. Shown is a representative experiment, repeated in triplicate.

**Figure 11:**
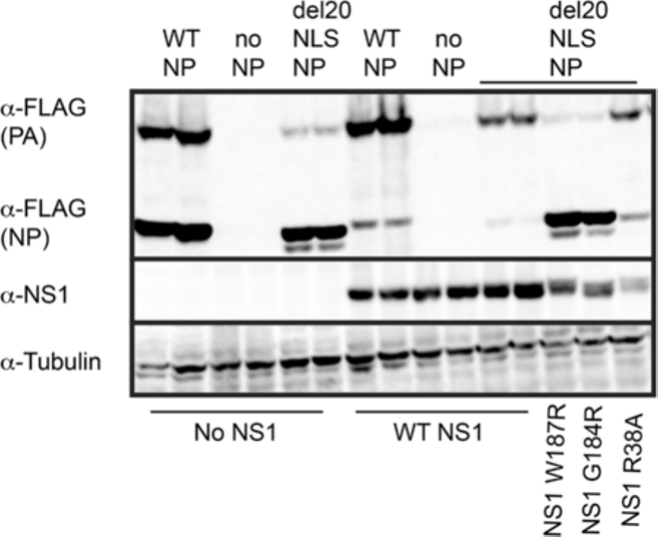
**NS1 RNA binding is not required for rescue but CPSF30 interaction domain is essential.** 293T cells were transfected with plasmid mixes to express recombinant vRNPs containing influenza RdRP with either WT-NP, del20NLS-NP, or no NP, and FLAG-PA vRNA as template in the presence or absence of NS1 and NS1 mutants as indicated. Total protein was separated by SDS-PAGE and analyzed by Western blotting with anti-FLAG antibody to visualize FLAG-PA products. Anti-NS1 detects NS1. Anti-tubulin detects tubulin and serves as a loading control. Shown is a representative experiment, repeated in triplicate.

To further verify that NS1 masks the gene expression defect observed in the presence of del20NLS-NP through enhancement of influenza mRNA translation, as opposed to facilitating formation of functional vRNPs or stimulating RNA synthesis, we examined the ability of NS1 to alleviate the gene expression defect observed with the GFP-M vRNA template. With GFP-M vRNA template, no RNA and protein were detected in the presence of del20NLS-NP, in contrast to FLAG-M vRNA template, where RNA and proteins products were reduced but clearly present (compare Figure 6 with Figure 4). If functional vRNP formation were stabilized or RNA synthesis were enhanced by the presence of NS1, we would expect to observe at least some alleviation of the gene expression defect at the protein level due to increased mRNA expression. However, if translation of existing mRNAs were enhanced, we would not expect to observe increased GFP-M protein expression, as no GFP-M mRNAs would be present for NS1 to act on and enhance translation. Indeed we observe the latter, with NS1 addition resulting in increased FLAG-M protein expression but not GFP-M protein expression (Figure 10). This data confirms our conclusion that NS1 masks the gene expression defect observed in the presence of del20NLS-NP via its ability to enhance viral mRNA translation [34, 35]. NS1 also results in increased PA protein expression, demonstrating the phenomenon is not restricted to M protein expression (Figure 11). The FLAG-PA produced from the vRNA template is not functional in reconstituted vRNP assays (unpublished observation, LLN) and thus cannot contribute to vRNP activity.

### NS1-CPSF30 interaction surface is required to enhance viral translation

To demonstrate the utility of del20NLS-NP to characterize the effect of additional proteins on influenza gene expression, we analyzed an NS1 RNA binding mutant and two NS1 CPSF30 binding site mutants. We found that expression of wild type NS1 or RNA binding deficient NS1 protein, NS1 R38A, resulted in increased FLAG-PA protein expression in the presence of del20NLS-NP (Figure 11). This is despite decreased NP and del20NLS-NP expression in the presence of both wild type NS1 and NS1 R38A due to NS1 interaction with CPSF30 and subsequent inhibition of cellular mRNA processing [38]. Expression of NP (and the polymerase subunits) in the reconstituted vRNP system is driven by host RNA polymerase II (Figure 1) and dependent on CPSF30 for proper 3’ end processing of NP (and polymerase subunit) encoding mRNAs. Our results are consistent with NS1 activity of viral translational enhancement, which does not require NS1 RNA binding [35] and prove activities of NS1 that require RNA binding, such as inhibition of RNase L [39] or interaction with host RNA helicase A [40], are not required to observe increased FLAG-PA protein expression in the presence of del20NLS-NP. While NS1 RNA binding is dispensable for increased FLAG-PA protein expression in the presence of del20NLS-NP, NS1 mutants in the CPSF30 interaction domain, NS1 W187R and NS1 G184R, do not result in improved protein expression of FLAG-PA (Figure 11), this is despite the increase in del20NLS-NP expression compared to samples expressing wild type NS1 or NS1 R38A proteins. Increased expression of del20NLS-NP proteins are expected, as NS1 G184R and NS1 W187R mutants are unable to inhibit cellular 3’ end processing. These results reveal that the CPSF30 binding domain of NS1, required to inhibit cellular mRNA polyadenylation in the nucleus, is essential for enhanced viral translation, an activity that occurs in the cytoplasm. Future experiments will address whether interaction with CPSF30 is required or if this NS1 interaction surface is involved in other important interactions. We speculate the latter will likely prove to be the case as translation occurs in the cytoplasm, while CPSF30 resides in the nucleus. Further, there is evidence that this same NS1 interaction surface is involved in viral virulence by an unknown mechanism that does not involve the IFN system [41]. Our results suggest this NS1 interaction surface is not only an antiviral target in the nucleus [42], but inhibition in the cytoplasm should also prove effective.

## Conclusion

We have two main conclusions. First we validate the significance of the N-terminus of NP aside from vRNP and NP nuclear import [20–22]. We did this by replacing the N-terminus of NP with the conventional NLS from SV40-T antigen to generate a nuclear localized N-terminal NP deletion mutant. Using reconstituted vRNP assays, we characterized del20NLS-NP, which maintained nucleotide binding but displayed diminished influenza RNA expression, exacerbated by vRNA template length. 1D blue native gel electrophoresis of total protein extracts from transfected 293 T cells expressing reconstituted vRNPs with GFP-NS1ss vRNA templates found little del20NLS-NP in higher molecular weight complexes, compared to WT-NP. Our results support a defect in functional vRNP formation with del20NLS-NP, resulting in decreased RNA and protein expression from the vRNA template. From characterization of this nuclear localized NP mutant we can conclude the N-terminus of NP plays a role in functional vRNP formation, independent of any role in vRNP nuclear localization.

The second conclusion asserts the usefulness of del20NLS-NP as a tool to characterize regulation of influenza gene expression. The fact that this NP mutant displays partial function facilitates study of the influence of NS1, NS2/NEP, and other factors on influenza RNA and protein expression. Unlike WT-NP, del20NLS-NP provides clear observable differences in viral protein expression in the presence or absence of additional factors in reconstituted vRNP assays. This conclusion is underscored by our confirmation that NS1 functions to enhance influenza mRNA translation [34, 35]. Further, NS1 mutant analysis reveals the NS1-CPSF30 interaction surface is important for this cytoplasmic function of NS1 and could prove an effective cytoplasmic antiviral target.

## Methods

### Cells

Human embryonic kidney (293 T) cell line was purchased from ATCC and maintained in a water-jacketed incubator at 37°C with 5% CO_2_ output.

### Plasmids

pcDNA plasmids expressing Influenza A Udorn mRNA to encode PA, PB1, PB2, NP, NS, NS1ss, NS1ss R38A, NS1ss W187R, NS1ss G184R proteins, and pHH21 plasmids expressing untagged M, HA, PA, PB1, and NS vRNAs, were gifts from the Krug laboratory. The pcDNA plasmid expressing WT-NP encoding a C-terminal Flag epitope tag was constructed using PCR (primers from Operon). In addition to proper restriction enzymes sites, the 5’ primer encoded the start codon and the first amino acids of NP while the 3’ primer encoded the last amino acids of NP followed by a short glycine linker and the FLAG epitope tag (DYKDDDDK) prior to the stop codon. The del20NLS-NP was constructed in a similar manner using the same 3’ primer but in this case the 5’ primer encoded the conventional NLS from SV40 T antigen (PKKKRKV) in between the start codon and NP codon 21. The PCR products were digested with EcoRI and XbaI and ligated into pcDNA vector. pHH21 Flag-NS vRNA, Flag-M vRNA, GFP-M vRNA, GFP-NS vRNA, GFP-NS1ss vRNA and Flag-PA vRNA, were constructed by two step PCR. The first step resulted in two DNA fragments, one encoding the antisense for the 5’ UTR–start codon and tag (FLAG or GFP) fused with the 5’ portion of the viral gene, the second encoding an overlapping region of the 5’ portion of the viral gene through the 3’ end. The two DNA fragments were used as templates in the second PCR step with BsmB1 5’common UTR and BsmB1 3’common UTR primers to produce the entire fusion coding sequence. The DNA insert was digested with BsmB1 and inserted into pHH21 so that the vRNA was expressed from the plasmid. The pHH21 PB1- GluGlu vRNA plasmid was made in a similar fashion except the tag was engineered at the 3’ of the PB1 gene. The FLAG-M short vRNA plasmid was made by digestion of the pHH21-FLAG-M vRNA plasmid with NcoI and ligation of the larger DNA fragment, resulting in a construct missing ~250 nucleotides within the intron sequence. All newly constructed plasmids were sequenced at the CSUPERB Sequencing Facility at San Diego State University to confirm successful plasmid construction.

### Antibodies

Anti-Udorn and anti-NS1 antibody were kind gifts from the Krug laboratory and used at a 1:1000 dilution for Western blotting. Anti-FLAG antibody was purchased from Stratagene and used as per manufacturer’s suggestion. Anti-Glu-Glu, anti-Nxf1, anti-Hsp90, and anti-Tubulin were purchased from Abcam and used as per manufacturer’s suggestion. Secondary HRP coupled anti-Rabbit, anti-Mouse, and anti-Goat were purchased from Pierce and used at a 1:10,000 dilution.

### DNA transfection

293 T cells were grown to approximately 70% confluency in either 10 cm or 6-well plates depending on experiment. Plasmid DNA was purified using Qiagen QIAprep spin plasmid purification kits as per the manufacturer’s protocol. DNA with pcDNA plasmids to express either WT-NP, del20NLS-NP, or vector, alone or in the presence of PB1, PB2, PA, and along with the appropriate pHH21 plasmid(s) to express vRNA templates, was mixed with Mirus transfection reagent at a 1:3 ratio of DNA to reagent and cells were transfected as per the manufacturer’s protocol. 48 hours post transfection cells were washed with 1X PBS, collected, and pelleted by centrifugation.

### Isolation of cytoplasmic and nuclear proteins

Cell pellets were washed twice with 5 volumes of the cell pellet in Reticulocyte Standard Buffer (RSB: 10 mM Tris HCl pH 7.5, 10 mM KCl, 1.5 mM MgCl_2_). Cells were then resuspended in RSB at 10× the volume of the cell pellet and incubated on ice for 10 minutes. NP-40 was added at a final concentration of 0.2 % to disrupt plasma membranes. Visual inspection of the cells before and after addition of NP-40 ensured that the plasma membranes were disrupted, but that the nuclei remained intact. Nuclei were pelleted by centrifugation at 300 *g* for 8 minutes at 4°C. The cytoplasmic extract was collected and the nuclear pellet was resuspended in Dignam buffer C without glycerol (20 mM HEPES pH 7.9, 0.42 M NaCl, 1.5 mM MgCl_2_, 0.2 mM EDTA) to release nuclear molecules. Both cytoplasmic and nuclear extracts were clarified from debris by high-speed centrifugation for 10 minutes at 4°C. An equal amount of 20 mM HEPES pH 7.9, 0.2 mM EDTA was added to the nuclear extract to reduce the total NaCl and MgCl_2_ concentrations. All buffers used contained protease inhibitors (Pierce).

### Isolation of total protein and RNA

To isolate total protein extracts, cell pellets were resuspended in 10 volumes of the cell pellet in RIPA Lysis Buffer (25 mM HCl pH 7.6, 150 mM NaCl, 1% deoxycholate, 0.1% SDS) with protease inhibitors. Cells were lysed and genomic DNA sheared using Fisher Scientific Sonic Dismembrator for 10 pulses at 30%, output 3-4.

Total RNA was isolated using Trizol (Invitrogen) according to the manufacturer’s protocol. RNA was quantified and evaluated for purity by taking OD_260_ and OD_280_ readings in duplicate on the NanoDrop ND1000 Nanospectrophotometer (Thermo Fisher Scientific).

### SDS-PAGE and Western Blot analysis

Protein extracts were separated by SDS-10%PAGE. Proteins were transferred to nitrocellulose using a Fisher semi- dry blot apparatus and probed with primary and HRP-conjugated secondary antibodies as indicated. Pierce ECL reagents were used to detect HRP conjugated secondary antibody. Blots were developed using the Chemi-Hi setting on the ChemiDoc XRS (BioRad) System and digital images were obtained using Quantity One software.

### GFP detection

GFP was visualized with a Nikon Eclipse TS100 (Nikon Intensilight C-HGFI for fluorescence) inverted microscope and images captured with the Nikon DS-Qi1Mc camera with NS Elements software.

### Reverse transcription – PCR

Prior to reverse transcription RNA OD was taken in duplicate to ensure use of equal concentrations of total RNA, and RNA was analyzed by gel electrophoresis to ensure no RNA degradation. RNA was first treated with RNase-free DNase (Promega) to degrade any contaminating plasmid DNA. 1 μg total RNA was subject to reverse transcription using oligo dT. Promega reverse transcription system was used as per the manufacturer’s protocol with the modification of increasing the reverse transcription time to 59 minutes to facilitate complete first strand cDNA synthesis. Reverse transcription reactions were aliquot from a master mix to ensure all samples received equivalent AMV-RT enzyme. Primers used in semi-quantitative PCR were as follows: M detection: (5’M) GCCTTCTGACCGAGGTCG, (3’M) CGATCAAGAATCCAC NS detection: (5’NS) GGATTCCAACACTGTG, (3’NS) CGATGTTTAGACCG HA detection: (5’HA) GTCGCTCTGGAGAACCAACATACAA, (3’HA) ACAAGGGTGTTTTTAATTACTAATA PB1-Glu-Glu detection: (5’PB1) GGGAAAGGATACATGAACGAAAGT, (3’GluGlu) CTCCATTGGCATGTACTC PCR products were separated on 1% agarose gels. Gels were analyzed with the UV setting on the ChemiDoc™ XRS (BioRad) System and digital images were captured using Quantity One software.

### Isolation of total protein extract for native gel and IP assays

Cell pellets were resuspended in 1 mL of sonication buffer (10 mM Tris HCl pH 7.5, 100 mM NaCl, 2.5 mM MgCl_2_, 0.5% Triton X-100). Cells were lysed using a Fisher Scientific Sonic Dismembrator for 30 pulses at 30%, output 3-4. Sonicated materials were loaded onto 1 mL of 30% sucrose cushion (30% sucrose, 10 mM Tris HCl pH 7.5, 10 mM NaCl, 2.5 mM MgCl_2_) and centrifuged at 4000 *g* for 15 minutes at 4°C to clarify the total protein extract. Total protein extract was used for 1D blue native gel electrophoresis or immuno purification.

### Immuno purification

Total protein extracts were incubated with anti-FLAG antibody (Stratagene) (1:50) for 1 hour at 4°C at which point PA/G sepharose beads washed in sonication buffer with protease inhibitors were added and incubation was continued at 4°C overnight. The samples were then spun for 1 minute at 1000 *g* at 4°C. For gel shift assays beads were washed 6 times. After washes in sonication buffer, beads were resuspended in 0.00125 mM 3X FLAG Peptide and incubated overnight at 4°C. Samples were then centrifuged at 1000 *g* for 1 minute. Supernatant, containing purified proteins, was collected. Samples were separated by SDS-PAGE and stained with Coomassie Blue.

### Gel shift

Purified protein preparations were incubated with 50 nucleotide long single stranded DNA modified with 5’ biotin for detection, and corresponding to the sequence of the 5’ and 3’ ends of the viral RNA (Biotin-AGCAAAAGCAGGGTGACAAAGACATGATAAAAAACACCCTTGTTTCTACT). After the incubation period the complexes were separated on a TBE (native) 8% PAGE, transferred to a nitrocellulose membrane using a Fisher semi-dry blot apparatus, and biotin labeled DNA was detected by incubation of the membrane with HRP conjugated streptadivin. Pierce ECL reagents were used to visualize HRP conjugated streptadivin. Blots were developed using the Chemi-Hi setting on the ChemiDoc™ XRS (BioRad) system and digital images were obtained using Quantity One software.

### 1D blue native gel electrophoresis

Total protein extracts were separated on 6% PAGE TBE (native) facilitated by coomassie blue [40]. Gel was run at 4°C until denatured molecular markers of at least 72 kDa ran off the gel. Further characterization is required to obtain the exact size(s) and molecular identity of other components present in the WT-NP containing higher molecular weight complexes observed. Proteins were transferred to nitrocellulose using a Fisher semi-dry blot apparatus. Blots were probed with primary and HRP-conjugated secondary antibodies as indicated. Pierce ECL reagents were used to detect HRP conjugated secondary antibody. Blots were developed using the Chemi-Hi setting on the ChemiDoc™ XRS (BioRad) system and digital images were obtained using Quantity One software.

## Authors’ information

AS was a Master’s graduate student, currently an instructor and laboratory technician at Moreno Valley College, Riverside Community College District, California. CFGJ was a MARC (Minority Access to Research Careers) undergraduate scholar, now a doctoral student at the University of California, Irvine. JR is a current MARC undergraduate scholar. All student researchers performed this work in the CSUSB laboratory of LLN.
